# Self-Assembled Hybrid Halide Perovskite Quantum Wire Bundle/Dot for Multiband Applications

**DOI:** 10.3390/nano14171443

**Published:** 2024-09-04

**Authors:** Hee Chang Jeon, Seonghwan Kim, Young-Seong Kim

**Affiliations:** 1 Quantum Functional Semiconductor Research Center, Dongguk University, Jung-gu, Seoul 04620, Republic of Korea; hcjeon@dongguk.edu; 2 Department of Mechanical and Manufacturing Engineering, Schulich School of Engineering, University of Calgary, Calgary, AB T2N 1N4, Canada; sskim@ucalgary.ca; 3 Department of Mechanical, Robotics and Energy Engineering, Dongguk University, Jung-gu, Seoul 04620, Republic of Korea

**Keywords:** quantum wire bundle/quantum dot, multiband applications, halide perovskite, photoluminescence

## Abstract

In this study, self-assembled halide perovskite quantum wire bundles (QWBs)/quantum dots (QDs) are fabricated using a room temperature-based formation method. The one-dimensional (1D) perovskite-based QWB structures incorporate zero-dimensional QDs within a composite quantum structure. Transmission electron microscopy reveals that quantum wires with diameters ranging from tens of nanometers to approximately 200 nm maintain a single-crystal atomic arrangement in a bundle form. Conversely, QDs are uniformly distributed within the single-phase wire and appear as black dots < 10 nm. Photoluminescence analysis identifies the multiband characteristics of the emissions. The 420–440 nm band is attributed to 1D QWB, whereas the peak appearing in the 530–550 nm range corresponds to lead halide PbBr_2_ QDs. Thus, the proposed self-assembled 1D QWB/QD composite structure exhibits novel multiband physical properties in the 420–440 and 530–550 nm bands; it offers new opportunities for designing materials with potential applications in optoelectronic devices.

## 1. Introduction

Most sterilizing wavelengths from the Sun that reach the Earth are in the blue wavelength range, whereas ultraviolet (UV) radiation is mostly absorbed by the ozone layer. In contrast to blue light, UV is an effective sterilant; however, UV-C can damage DNA, potentially causing cancer. UV-B is known to cause burns and other forms of skin cancer, and UV-A is associated with risks, such as wrinkles and skin aging [1,2,3]. Additionally, blue light from the Sun that reaches the Earth exhibits antimicrobial and antiviral properties [4,5]. Simulations have shown that the influenza virus is inactivated by sunlight, which supports the hypothesis that the sterilizing ability of the Sun is due to radiation in the blue wavelength range [4,6]. Antiviral and germicidal effects have been observed at 420–430 nm wavelengths, although enveloped viruses are unaffected by radiation in this range. Violet–blue light is known to damage viral nucleic acids [5]. Enveloped viruses are more sensitive to photodynamic inactivation than non-enveloped viruses [7,8,9]. Materials with dual-energy band characteristics respond to both UV and visible light and are highly valuable for air purification owing to their wide surface area and photocatalytic activities [10]. Currently, GaN (gallium nitride)-based light-emitting diodes (LEDs) are extensively used for UV-bluish LEDs despite several limitations, such as the efficiency droop phenomenon, where their efficiency decreases with an increase in current [11].

Hybrid halide perovskites are used in various blue-light applications owing to their excellent optoelectronic properties, high efficiency, and competitive pricing. The exceptional optoelectronic characteristics of perovskites are attributed to their high light-absorption coefficient, balanced electron–hole transport, narrow full width at half maximum in photoluminescence (PL), high PL quantum yield, easily tunable spectrum based on the chemical composition, and high charge mobility [12,13,14]. These properties have resulted in the increasing use of perovskites as emissive layers in LEDs [15].

Hybrid organic–inorganic perovskite materials with varied organic and inorganic chemical compositions have been extensively investigated owing to their ability to exhibit a wide range of optical properties across different wavelengths. The field of light-emitting devices has witnessed substantial advances in simplifying the fabrication process of perovskites to achieve superior optical properties. Substituting iodine with chlorine or bromine can adjust the wavelength from UV to near-infrared. Extensive research has been conducted on colloidal perovskite nanostructures. The discrete changes in luminescent energy based on the nanostructure have also been investigated [16]. Additionally, the optical properties of photoluminescence in materials vary depending on their heterostructure [17,18,19,20]. Considering the beneficial characteristics of perovskites, research utilizing quantum structures as materials for UV-bluish LEDs has garnered interest. In particular, the favorable optical properties of perovskites have drawn attention to the study of quantum wire structures [21,22].

This study explores the structural characteristics of self-assembled quantum wire bundles (QWBs)/quantum dots (QDs) of halide perovskites using high-resolution transmission electron microscopy (HR-TEM). A synthesis method is developed to demonstrate the UV-bluish-emitting characteristics through PL in an aprotic solution at room temperature (300 K). This approach does not involve the mere substitution of iodine groups, the use of hetero-bivalent ions, or the reduction in layers. Perovskite materials featuring a hybrid structure of one-dimensional (1D) QWB and zero-dimensional (0D) quantum dots (QDs) are developed with desirable physical properties in the 420–440 and 530–550 nm bands.

## 2. Materials and Methods

### 2.1. Materials

The compound (C_4_H_9_NH_3_)_2_PbI_2_Br_2_ was synthesized using a chemical method with the following reagents: N-butylamine 99+% (C_4_H_9_NH_2_), hydriodic acid (47%) with 1.5% hypophosphorous acid, lead (II) bromide, 98+% extra pure anhydrous diethyl ether (stabilized with butylated hydroxytoluene), ethyl alcohol (99.5%), dimethyl sulfoxide (DMSO) (99.9% min), and N,N-dimethylformamide (DMF) (99.8%). N-butylamine and lead bromide were obtained from ACROS, hydroiodic acid, and DMSO from Alfa Aesar, diethyl ether from TCI, and DMF and ethyl alcohol from Daejung Chemicals.

### 2.2. Methods

#### 2.2.1. Precursor Synthesis

To synthesize the precursor, ethanol and 47 wt.% hydroiodic acid were first combined in a beaker, following which N-butylammonium was added while stirring. The mixture was stirred for 30 min, reacted in an oil bath at 70 °C for 2 h, and subjected to vacuum treatment at 90 °C for 20 min in a vacuum oven. This produced a white powder, which was washed three times with ether using a vacuum filtration system and vacuum-dried at 70 °C for 12 h.

#### 2.2.2. Synthesis of Perovskite Wire Crystal Structure

For synthesizing the perovskite wire crystal structure, a solvent mixture of DMF and DMSO in a 1:1 volume ratio was prepared in a vial. Subsequently, C_4_H_9_NH_3_I precursor and PbBr_2_ were added in a 1:1 molar ratio at a concentration of 0.5 M. Within a day, needle-shaped flower crystals started to form, eventually growing into white-colored, wire-shaped crystals after being left at room temperature (300 K) for three days, filling the vial.

### 2.3. Characterization

X-ray diffraction (XRD) analysis was performed using an X’Pert PRO MPD instrument (Almelo, The Netherlands) equipped with a Cu radiation source. HR-TEM measurements were taken with an FEI Tecnai instrument (Hillsboro, OR, USA) at an operating voltage of 300 kV. PL analysis was performed using a LabRAM HR Evolution instrument (Kyoto, Japan) from HORIBA, featuring a charge-coupled device detector and a 325 nm laser. UV–Vis measurements were performed using a Cary 5G optical spectrometer (Santa Clara, CA, USA) at the Daegu Center of the Korean Basic Science Institute. X-ray photoelectron spectroscopy (XPS) measurements were performed using an AXIS SUPRA instrument (Kyoto, Japan) from Kratos at the National Center for Inter-University Research Facilities.

## 3. Results and Discussion

**Figure 1:**
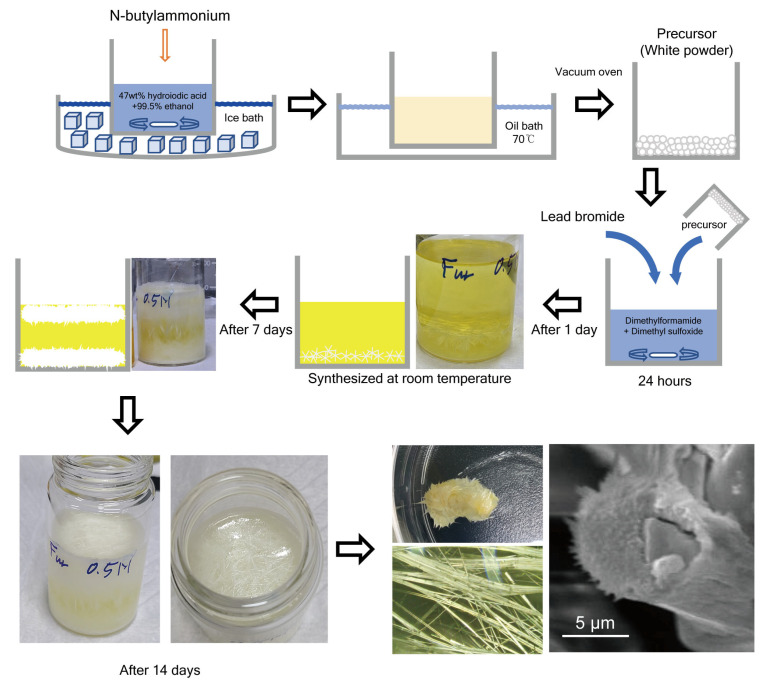
Fabrication method of the self-assembled hybrid halide perovskite QWB/QD. The bundled shape of the wires is evident in the SEM images.

A mixed-halide perovskite quantum structure exhibiting two-band blue-emitting properties was developed using a novel room-temperature synthesis method. The synthesized self-assembled QWB (Figure 1) comprised quantum wires (QWs) with diameters in the range 25–150 nm. The QWs were aligned in the same direction, forming a bundle structure and confirming the QWB perovskite formation. The QDs were not detected by scanning electron microscopy (SEM), thus necessitating TEM measurements.

**Figure 2:**
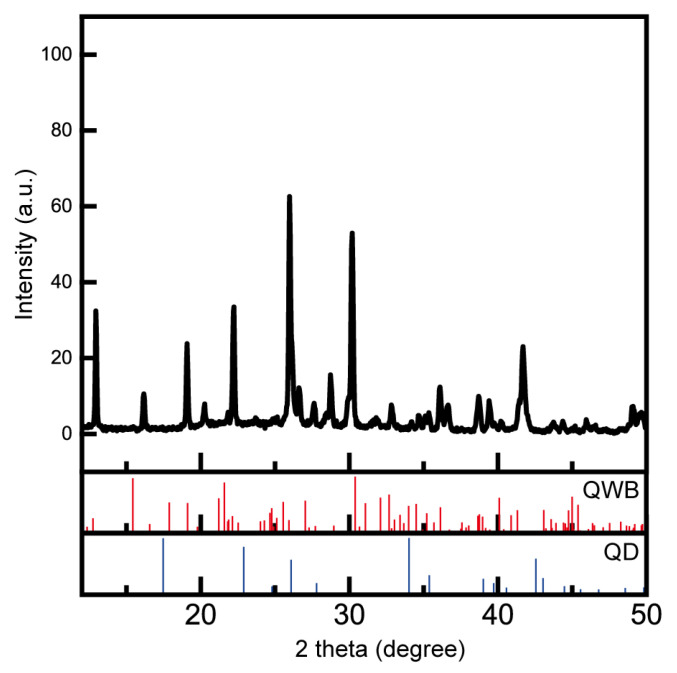
XRD analysis showing the peaks of QWB and QD, confirming the hybrid structure. The simulated XRD peak results obtained through material modeling have been included alongside the experimental data.

Figure 2 shows the XRD results for the QWB/QD structure, revealing the coexistence of XRD peaks corresponding to the QWB perovskite structure BAPbI_2_Br_2_, along with those of the QD structure from lead halide clusters. By matching these peaks through simulation, we can identify the corresponding material for each peak. A comparison with previously published XRD peaks indicated agreement with BAPbI_2_Br_2_ [14,23], closely aligning with the calculated XRD patterns. Some additional peaks are likely due to heavy metal and organic impurities that form during the synthesis process, which can arise depending on the solvent growth method and the reaction progress of the precursors [24]. The remaining peaks corresponding to the lead halide clusters aligned with the simulated XRD pattern of PbBr_2_, indicating that the wire-shaped perovskite and dot-shaped lead halide clusters formed a composite of BAPbI_2_Br_2_ and PbBr_2_ within the QWB/QD structure. The formation of these structures was attributed to the room-temperature synthesis method used in this study, in which an increase in DMSO resulted in enhanced coordination between the solution and lead halide. This promoted the formation of lead halide clusters within BAPbI_2_Br_2_ and subsequently induced the formation of QDs [25].

**Figure 3:**
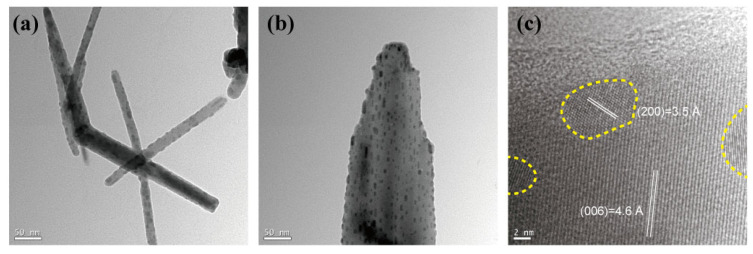
TEM images of QW and QD: (**a**,**b**) TEM images of QW and QD, respectively; (**c**) HR-TEM image confirming both structures. The region containing QD in QW is demarcated by a yellow dashed line.

The TEM images (Figure 3) revealed the formation of QWs with diameters ranging from tens of nanometers to approximately 200 nm, which maintained a single-crystal atomic arrangement in a bundled form (Figure 3a,b). The internal structure of the wires and the formation of QDs were identified. The simulated XRD results conducted based on modeling performed using Mercury software (2020.3.0) confirmed that the phase of BAPbI_2_Br_2_ is orthorhombic. The QWB phase of BAPbI_2_Br_2_ is orthorhombic, and the interplanar spacings (d = 4.6 Å) and crystal lattice parameters (c = 27.6 Å) were identified and marked through TEM analysis. Similarly, for QDs, the phase was confirmed to be tetragonal, and the interplanar spacings (d = 3.5 Å) and crystal lattice parameters (a = 7.0 Å) were calculated and validated through TEM results (Figure 3c). Structurally, the wires formed as single-phase structures, and the QDs, appearing as black dots < 10 nm, were uniformly distributed (Figure 3). Thus, the coexistence of two different physical properties within the single structure of the QWs, owing to the presence of QDs embedded within them, was confirmed. This phenomenon occurred when QDs were incorporated into quantum wires during formation, resulting in the creation of multiple bands depending on their size [26].

**Figure 4:**
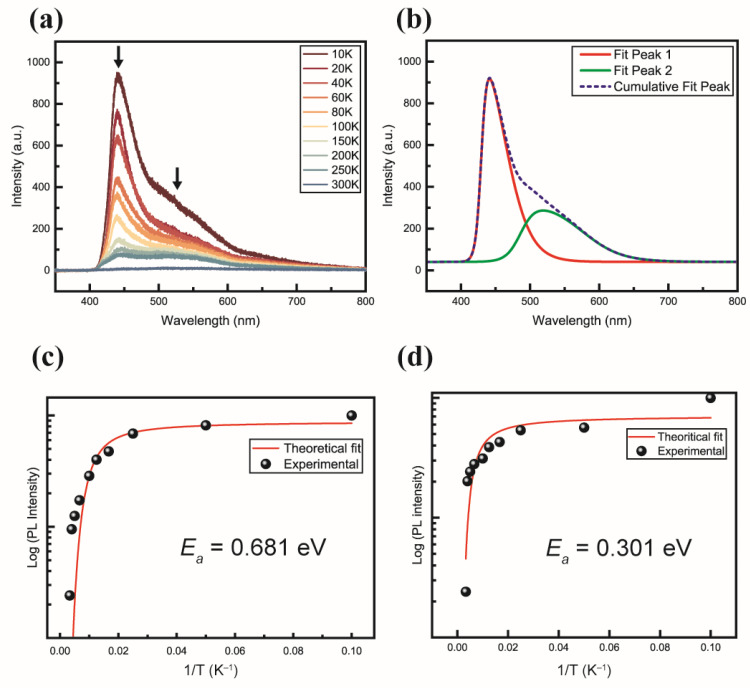
Results of the photoluminescence analysis: (**a**) temperature dependence of PL, (**b**) outcomes of a multi-peak analysis of PL conducted at 10 K, calculation of the activation energy using the Arrhenius equation for the wavelength ranges of (**c**) 420–440 nm and (**d**) 530–550 nm.

The PL results (Figure 4) revealed the temperature-dependent behavior of the PL peaks. With increasing temperature, the peaks in the 420–440 and 530–550 nm bands exhibited a red shift, which is a characteristic of exciton peaks (Figure 4a). This temperature dependence indicates the band-shrinking phenomenon of the peaks in the 420–440 and 530–550 nm regions (Figure 4b), with activation energies calculated to be 0.681 and 0.301 eV, respectively (Figure 4c,d). The peak intensities of the bands in the 420–440 nm and 530–550 nm ranges increased with the excitation power. This confirmed that the behavior of the two bands aligned with that of the exciton peaks, as evidenced by their responses in the temperature-dependence experiments.

Nanosized wires and dots, through the quantum confinement effect, exhibited a blue shift toward violet-blue light characteristics, surpassing those of conventional thin-film structures for both bands. Previous studies on nanostructures have shown that BAPbI_2_Br_2_ exhibited a single peak at 420 nm [23], and lead halide PbBr_2_ exhibited PL characteristics in the 520 nm band [17]. The band structure is influenced by factors such as the interface effect. These effects contribute to the electronic transitions and carrier transport behavior, broadening the PL peak from the 0D/1D structures [18,19,20]. This study revealed that the origin of the 420 and 520 nm PL peak bands in a composite structure of BAPbI_2_Br_2_ QWB with embedded PbBr_2_ QDs can be attributed to the formation of two optical energy bands, owing to the presence of the QDs within the QWB. This suggests the coexistence of two different optical energy bands in the material. The activation energy of the 1D perovskite QWB in the 420–440 nm band was measured to be 0.681 eV, and that of the PbBr_2_ QDs in the 530–550 nm range was 0.301 eV (Figure 4c,d). Thus, the observed optical properties of the composite are strongly correlated with its structural composition.

Based on these findings, the 420–440 nm band in the self-assembled 1D QWB/QD composite structure originated from the 1D QWB. Similarly, the peak observed in the 530–550 nm range was attributed to the QDs of lead halide PbBr_2_. This distinction underscores the role of the composite structure in producing specific optical characteristics, wherein the integration of quantum wires and dots within the same material results in distinct PL peaks in these two wavelength bands.

**Figure 5:**
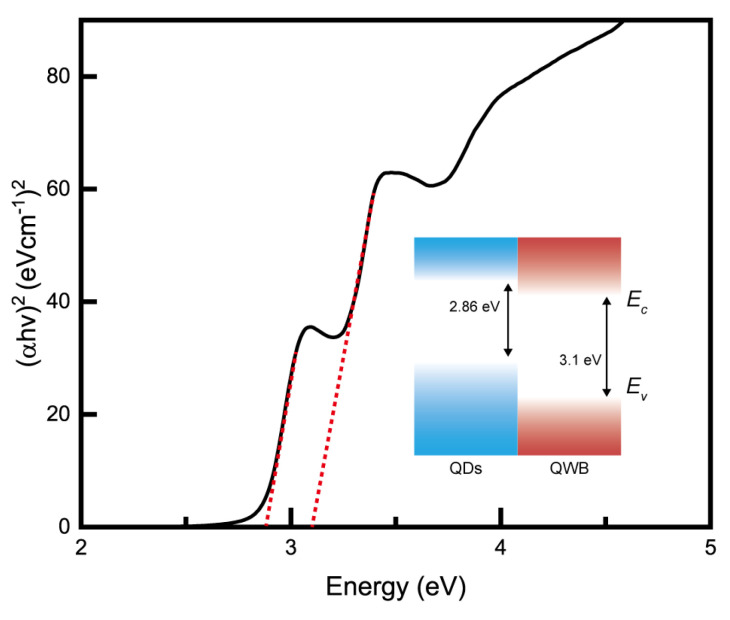
UV–Vis absorption data represented using a Taut plot confirmed the presence of bandgaps at 2.86 and 3.1 eV.

The UV–Vis spectroscopy data are presented in Figure 5. The self-assembled QDs/QWB composite structure exhibited energy band gaps measured at 2.86 and 3.1 eV (inset Figure 5). A comparison of these values with the PL results facilitated a more accurate confirmation of the coexistence of two distinct optical energy band structures within the nanoscale architecture. This alignment between the UV–Vis absorption and PL emission data further validated the presence of specific energy band gaps corresponding to the unique quantum mechanical properties of the hybrid structure, demonstrating the effectiveness of the composite for targeted optical applications.

The coexistence of the two nanostructures, namely the 1D QWB mixed-halide perovskite phase, and QDs, was attributed to the room temperature-based synthesis of the composite. Specifically, lead halide nanoclusters crystallized in the presence of DMSO at room temperature [25]. This process underscores the role of solvent chemistry and environmental conditions in directing the nucleation and growth of distinct nanostructured phases within a single-material system, enabling the simultaneous development of QWBs and QD clusters. This innovative synthesis approach leverages the unique properties of DMSO as a solvent to mediate the crystallization of lead halide components, thereby creating a hybrid nanostructure with potential applications in optoelectronics, photonics, and energy-conversion devices.

**Figure 6:**
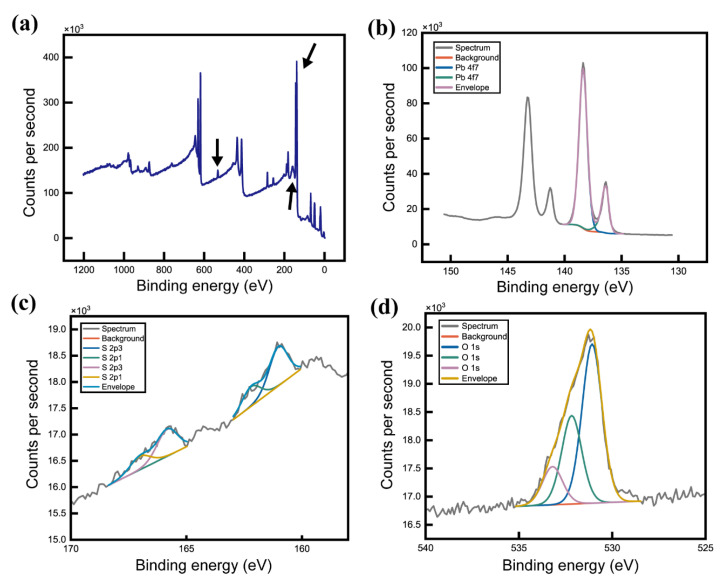
XPS spectra of (**a**) the wide scan spectrum for QWB/QD, (**b**) Pb 4f, (**c**) S 2p, and (**d**) O 1s.

The XPS results (Figure 6) show the presence of S and O atoms, which can be understood through the interaction of DMF and DMSO with the lead halides. Weak Pb-O bonds formed when DMSO was added to DMF, primarily because of the polarity of DMSO (Figure 6b–d). The S-O bonds lead to covalent bonding between DMSO and lead halide, facilitating the formation of a self-assembled, needle-shaped crystal structure [25]. This phenomenon was attributed to the aprotic nature of the solvent, which contained C=O or S=O bonds that interacted with Pb to form Pb-O bonds. Thus, the interaction between DMF, DMSO, and the precursor solution resulted in the formation of intermediate structures and, eventually, a complex nanostructure. This underlines the crucial role of solvent chemistry in directing the crystallization process, thus enabling the strategic manipulation of material morphology through solvent selection and precise control of the conditions for its synthesis.

To exploit the optical characteristics of multibands, the metals and halides were mixed to form a composite of bromine and chlorine in perovskites following a room temperature-based synthesis method, which led to phase separation and the formation of halide-based QWB/QDs. This approach was particularly effective for creating blue-light PeLEDs, reducing their dimensions by incorporating two different cations. Thus, a complex quantum structure was created, wherein 0D QDs were embedded in a 1D QWB perovskite base. A 1D QWB perovskite base has the advantage of enhanced hole migration compared with a 3D structure. This improves the optical and electrical properties and the performance of perovskite photovoltaics [27,28]. The synthesis and structural strategy presented in this study opens novel avenues for the design and development of advanced optoelectronic devices with enhanced performance and multifunctionality.

## 4. Conclusions

In this study, self-assembled halide perovskite QWBs/QDs were synthesized using a room temperature-based formation method. The composite had a complex quantum structure, wherein 0D QDs were embedded in a 1D QWB perovskite base. This structure exhibited multiband PL characteristics. Specifically, the 420–440 nm band originated from the 1D QWB, whereas the peak appearing in the 530–550 nm range was attributed to the QDs of the lead halide PbBr_2_. The distinct physical properties of the two bands suggest the potential for their application in multiphotonic devices.

## Data Availability

The data are contained within the article.
